# Antioxidant Activity of Natural Products: Recent Advances, Challenges, and Future Directions

**DOI:** 10.3390/antiox15050650

**Published:** 2026-05-21

**Authors:** José Pinela, Maria Inês Dias, Alexandra Plácido, Carla Pereira

**Affiliations:** 1 National Institute for Agricultural and Veterinary Research (INIAV, I.P.), Rua dos Lágidos, Lugar da Madalena, Vairão, 4485-655 Vila do Conde, Portugal; 2 CIMO, LA SusTEC, Instituto Politécnico de Bragança, Campus de Santa Apolónia, 5300-253 Bragança, Portugal; maria.ines@ipb.pt (M.I.D.); carlap@ipb.pt (C.P.); 3 LAQV/REQUIMTE, Departamento de Química e Bioquímica, Faculdade de Ciências, Universidade do Porto, Rua do Campo Alegre, s/n, 4169-007 Porto, Portugal; alexandra.nascimento@fc.up.pt

## 1. Introduction

Oxidative stress represents a fundamental paradox in biology. The controlled generation of reactive oxygen and nitrogen species (ROS/RNS) is essential for vital physiological processes, including cellular signalling, immune defence, and mitogenic responses. However, their excessive production initiates a cascade of molecular damage. When the cell’s endogenous antioxidant network, comprising enzymes such as superoxide dismutase, catalase, and glutathione peroxidase, alongside low-molecular-weight antioxidants such as glutathione, becomes overwhelmed, a state of redox imbalance ensues. This imbalance leads to lipid peroxidation, protein carbonylation and nitration, and oxidative DNA damage, progressively undermining cellular integrity and function [1,2]. Far from a mere biochemical epiphenomenon, oxidative stress is now widely recognised as a central pathogenic mechanism underpinning many of the most prevalent non-communicable diseases, including neurodegenerative disorders, cardiovascular disease, type 2 diabetes, and chronic inflammatory conditions, as well as the ageing process itself (contributions 1–5).

Confronted with this pervasive biological challenge, the scientific focus has evolved from the simple identification of radical scavengers toward the discovery of agents capable of restoring and maintaining systemic redox homeostasis. Natural products, shaped by evolutionary pressures over millions of years, constitute an unparalleled reservoir of chemically diverse and biologically active molecules. Far from representing pre-scientific remedies, they provide sophisticated molecular scaffolds for targeted redox modulation [3]. Polyphenols, organosulfur compounds, carotenoids, peptides, and other specialised metabolites frequently exhibit pleiotropic mechanisms of action that extend beyond direct radical scavenging to include modulation of endogenous antioxidant defences, regulation of redox-sensitive signalling pathways, and protection of cellular structures (contributions 1, 4, 6, and 7).

The Topic “Antioxidant Activity of Natural Products”, involving the journals Antioxidants, BioChem, Biomolecules, International Journal of Molecular Sciences, Marine Drugs, and Molecules, received 145 manuscripts for consideration, of which 34 were accepted for publication after a rigorous pre-check and peer-review process. These articles provide a comprehensive overview of this rapidly advancing interdisciplinary field, encompassing diverse natural sources of antioxidants, including plant metabolites (contribution 8), animal-derived peptides (contribution 9), and marine bioactive compounds (contributions 10 and 11), while exploring their biological effects across a range of experimental systems, from cellular models (contributions 6 and 7) to whole-organism studies (contributions 12–14). Together, these contributions demonstrate that research on natural antioxidants has moved beyond the descriptive cataloguing of in vitro activity, now emphasising mechanistic elucidation, biological validation, and translational relevance in health and disease.

## 2. Thematic Overview: From Sources to Application

**Figure 1:**
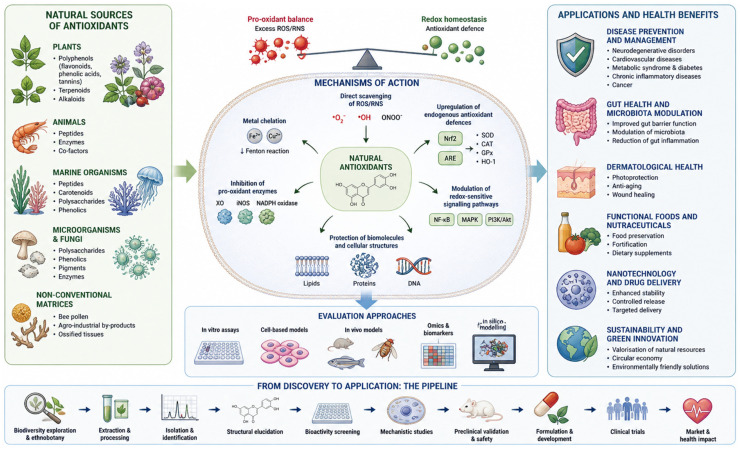
Integrated overview of sources, mechanisms of action, and health-related and technological applications of natural antioxidants. Major sources include plants, animals, and marine organisms, as well as non-conventional matrices. Antioxidants exert their effects through multiple mechanisms, including direct scavenging of reactive oxygen and nitrogen species (ROS/RNS), metal chelation, inhibition of pro-oxidant enzymes, and modulation of redox-sensitive signalling pathways (e.g., Nrf2, NF-κB, MAPK). These processes contribute to redox homeostasis and the protection of key biomolecules (lipids, proteins, and DNA). The figure also highlights experimental approaches for antioxidant activity evaluation and the research pipeline from discovery to application.

The contributions included in this Topic illustrate the broad and interconnected landscape of natural antioxidant research, spanning from source identification and chemical characterisation to mechanistic elucidation and practical application. Natural antioxidants originate from a wide diversity of biological matrices and exert their effects through multiple complementary mechanisms, ultimately contributing to the maintenance of redox homeostasis and cellular protection. A schematic overview of these relationships, including sources, mechanisms of action, evaluation strategies, and application domains, is presented in Figure 1. This integrative perspective reflects the current evolution of the field, in which multidisciplinary approaches converge to translate natural antioxidant potential into tangible health and technological outcomes.

### 2.1. Antioxidant Discovery and Chemical Characterisation

The diversification of natural antioxidant sources stands as a prominent theme, supported by advanced analytical techniques for detailed metabolite profiling. Several contributions move beyond traditionally studied natural products to explore unconventional and underutilised matrices, revealing previously unrecognised antioxidant potential. For instance, monofloral bee-collected pollen was shown to contain a complex phytochemical profile rich in phenolic acids, flavonoids, phenylamides, and alkaloids, with strong antioxidant activity demonstrated across multiple in vitro assays (contribution 15). In parallel, alternative marine- and animal-derived materials are also being increasingly investigated. Jellyfish-derived peptides (contribution 10), thioredoxin-based systems (contribution 11), and collagen-derived peptides from ossified antler tissue (contribution 9) were characterised as antioxidants with marked cytoprotective properties. These findings highlight the potential of non-conventional raw materials as reservoirs of bioactive compounds.

Plant-derived matrices remain central, but with increasing emphasis on chemical depth, compound isolation, and structural elucidation. Phytochemical analyses of species such as *Tectona grandis* L.f. (contribution 8), *Annona squamosa* L. (contribution 16), *Bougainvillea buttiana* Holttum & Standl. (contribution 17), *Sonchus brachyotus* DC. (contribution 18), and *Salix pseudolasiogyne* H.Lév. (contribution 19) enabled the identification of structurally diverse metabolites, several of which exhibited pronounced antioxidant properties alongside other relevant biological effects. In these studies, techniques such as LC-MS, GC-MS, HRMS, NMR, and in silico analysis were crucial for compound identification.

Several studies further extend this discovery framework by integrating sustainability-oriented approaches, particularly through the valorisation of agro-industrial by-products and underutilised biological resources. For example, olive pomace, an agro-industrial by-product of olive oil production, is characterised by a complex matrix rich in residual lipids, unsaturated fatty acids, and bioactive compounds such as tyrosol and hydroxytyrosol (contribution 20), highlighting its potential as a source of natural antioxidants and value-added lipid fractions. Complementarily, resource-efficient extraction methods further support this approach. Techniques such as ultrasound-assisted aqueous two-phase extraction enhanced the recovery and activity of natural antioxidants, including flavonoid glycosides from *Malvaviscus arboreus* Dill. ex Cav. flowers (contribution 21).

These articles demonstrate that antioxidant discovery is increasingly driven by the convergence of advanced analytical techniques and sustainability principles, in which compound separation and characterisation, by-product valorisation, and green processing converge to generate novel bioactive ingredients with multiple applications.

### 2.2. Biological Validation of Antioxidant Activity

The transition from in vitro chemical-based antioxidant activity assays to biologically relevant validation represents a key advance across the Topic contributions. Although methods such as DPPH, ABTS, FRAP, and ORAC remain useful for antioxidant activity screening, many studies adopt tiered approaches that integrate chemical characterisation with cellular and organismal models of oxidative stress. At the cellular level, some studies report cytoprotective effects in models exposed to oxidative insults (contributions 9, 11, 17, 22, and 23). Compounds such as baicalin, collagen-derived peptides, and jellyfish-derived thioredoxin reduced intracellular ROS levels, attenuated lipid peroxidation, and restored antioxidant enzyme activity, often through activation of signalling pathways such as AMPK/Nrf2 (contributions 6, 9, and 11). Mechanistic insights further reveal modulation of redox-sensitive signalling networks, including p38/Nrf2/HO-1, NF-κB, and ROS-JNK/MAPK signalling pathways, linking antioxidant activity to broader anti-inflammatory and cytoregulatory effects (contributions 2, 7, and 24). Other mechanisms have also been reported, such as inhibition of ferroptosis and apoptosis during myocardial ischaemia/reperfusion injury, exemplified by salvianolic acid B (contribution 2).

Some studies extend validation to whole-organism models. In *Drosophila melanogaster*, silibinin improved lifespan and functional performance (contribution 12), while in zebrafish (*Danio rerio*) larvae, compounds such as β-sitosterol reduced CuSO_4_-induced oxidative stress and inflammation (contribution 13). In a rat model of ethanol-induced gastric ulcer, ovothiol A demonstrated gastroprotective effects by reducing oxidative stress, inflammation, and apoptosis while enhancing antioxidant defences and preserving gastric mucosal integrity (contribution 25). Similarly, a study employing a non-conventional in vivo model of Alzheimer’s disease (*Globodera pallida*) demonstrates that plant-derived extracts can mitigate β-amyloid-induced toxicity by reducing ROS production, improving survival, restoring behavioural responses, and enhancing endogenous antioxidant systems (contribution 14). Further studies also support antioxidant effects across diverse biological systems and experimental models (contributions 26 and 27). These findings reveal that antioxidant activity is increasingly defined by the ability to modulate cellular redox homeostasis and confer measurable biological protection across multiple levels of complexity.

### 2.3. Mechanistic Insights and Integrated Modelling Approaches

A distinct subset of contributions focuses on elucidating the molecular mechanisms underlying antioxidant activity, combining computational and experimental approaches to resolve reaction pathways at a fundamental level. Computational methods, particularly density functional theory (DFT), enable the evaluation of thermodynamic and kinetic parameters governing antioxidant reactivity, including bond dissociation enthalpies, ionisation potentials, and proton affinities. These descriptors are critical for predicting dominant mechanisms such as hydrogen atom transfer (HAT), single-electron transfer followed by proton transfer (SET-PT), or sequential proton loss electron transfer (SPLET), which are highly dependent on the chemical environment. For instance, studies on isoflavonoids from *Astragalus mongholicus* Bunge demonstrate that HAT predominates in the gas phase or in non-polar environments, whereas SPLET becomes favourable in polar media, highlighting the decisive role of solvent effects in modulating antioxidant pathways (contribution 28).

Experimental techniques provide complementary mechanistic resolution by directly probing radical intermediates and transient species. Advanced approaches, such as chemically induced dynamic nuclear polarisation (CIDNP), reveal that antioxidant activity can involve not only radical scavenging but also interception of reactive electrons and modulation of radical chain reactions. For example, glycyrrhizin has been shown to capture solvated electrons, thereby preventing their interaction with molecular oxygen and altering downstream reactive oxygen species formation (contribution 29). Similarly, studies on compounds such as loureirin C demonstrate the coexistence of multiple reaction channels, including hydrogen abstraction, electron transfer, and radical adduct formation, with the dominant pathway depending on the reaction environment and energetic landscape (contribution 30). Collectively, these findings highlight that antioxidant effects arise from a network of competing and condition-dependent reaction mechanisms rather than a single dominant pathway.

At the biological level, these molecular mechanisms translate into the modulation of redox-sensitive signalling networks and cell fate pathways. Some studies demonstrate that antioxidants regulate key pathways such as P38/Nrf2/HO-1, which are tightly coupled to intracellular ROS levels and redox homeostasis (contribution 7). Beyond classical antioxidant responses, modulation of oxidative stress also affects mitochondrial function, apoptosis, and regulated cell death. For instance, inhibition of ROS-mediated necroptosis through suppression of RIPK3/MLKL signalling has been observed in intestinal models, illustrating how redox-active compounds can directly interfere with programmed cell death pathways (contribution 31). Other studies further extend this mechanistic framework to ferroptosis and mitochondrial dysfunction, reinforcing the concept that antioxidant activity is deeply embedded in cellular metabolic and signalling networks (contributions 2 and 5). In addition, interactions between antioxidants and biological macromolecules, including proteins and membranes, may influence their bioavailability, localisation, and functional efficacy (contribution 1), adding another layer of mechanistic complexity.

Taken together, these approaches bridge the gap between fundamental chemical reactivity and biological function, advancing the field from conventional antioxidant capacity measurements toward a mechanistic understanding of redox regulation. This integrative perspective is essential for the rational design of next-generation antioxidants and their translation into disease-preventive and therapeutic strategies.

### 2.4. From Improved Bioactivity to Application

Several studies demonstrate that compounds with strong radical-scavenging capacity in vitro may exhibit variable performance in complex matrices or biological environments, highlighting the context-dependent nature of their functionality. To address these limitations, some studies explore formulation and processing strategies to enhance stability and functional performance. For example, the one-pot enzymatic acylation of phenolic compounds (e.g., hydroxytyrosol) with olive pomace oil, catalysed by an immobilised lipase under solvent-free conditions, produced modified oils rich in structured phenolipids. These modified oils exhibited significantly improved oxidative stability and enhanced stability in food emulsions, as shown by a mayonnaise that remained stable throughout five months of storage, illustrating a clear pathway from agro-industrial by-products to functional food ingredients (contribution 20). Extraction technologies also play a critical role in determining functional outcomes, not only with regard to yield but also bioactivity (contribution 21).

Nanotechnology-based approaches further expand this perspective. Studies on zinc oxide nanoparticles synthesised with rutin or plant extracts demonstrate that nanoformulations can modulate biological activity and improve synthesis efficiency, even when the intrinsic radical-scavenging capacity is not markedly increased. Rutin-functionalised ZnO NPs exhibited potent anticancer activity and reduced environmental toxicity (contribution 32), while a systematic comparison of plant extracts showed that higher antioxidant activity of the extract leads to smaller, purer nanoparticles with higher yield (contribution 33). Together, these findings highlight a conceptual shift in antioxidant research. Functionality is now increasingly linked not only to chemical reactivity but also to bioavailability, cellular interactions, and its role in directing material synthesis, while biologically driven processes can offer more sustainable alternatives to conventional chemical methods.

Matrix-based formulations provide an additional layer of functional applicability. Hyaluronic acid-based systems incorporating bioactive components have demonstrated combined antioxidant, regenerative, and anti-ageing properties, including the stimulation of fibroblast and keratinocyte proliferation, migration, and extracellular matrix production. These systems also reduce intracellular oxidative stress, highlighting their capacity to modulate key cellular processes involved in skin ageing. Such integrated biomaterial platforms therefore hold significant potential for biomedical and nutraceutical applications (contribution 34). In parallel, studies on antioxidant–protein interactions indicate that binding phenomena can significantly influence distribution, stability, and biological efficacy in vivo, further emphasising the importance of system-level considerations.

## 3. Persistent Challenges and Future Directions

Despite the significant advances highlighted across the 34 contributions in this Topic, several important challenges remain. A persistent limitation is the lack of methodological standardisation in the assessment of antioxidant activity. The continued reliance on diverse chemical-based assays, often combined with heterogeneous cellular and organismal models, complicates direct comparison across studies and limits reproducibility. While many contributions move beyond simple in vitro assays, the variability in experimental design, endpoints, and reporting underscores the need for more harmonised and biologically relevant evaluation frameworks.

A major bottleneck remains the gap between promising preclinical findings and confirmed human benefit. Although numerous studies consistently report antioxidant, cytoprotective, and anti-inflammatory effects in cellular systems (e.g., keratinocytes, intestinal epithelial cells, macrophages) and in vivo models such as *Drosophila melanogaster*, zebrafish, and rodents (contributions 6, 12, and 13), robust clinical validation remains limited. Notably, within the contributions gathered in this Topic, only one randomised, double-blind, placebo-controlled clinical trial provides direct evidence in humans. This study evaluated a polyphenol-rich supplement containing *Pinus massoniana* bark extract in healthy older adults over 12 weeks and demonstrated reductions in oxidative stress biomarkers, particularly malondialdehyde, compared to a placebo (contribution 3). This highlights a critical translational gap and reinforces the need for well-designed clinical studies using standardised extracts, defined dosing regimens, and clinically meaningful endpoints.

The intrinsic complexity of natural products further complicates characterisation and application. The presence of diverse compound mixtures, in which synergistic or antagonistic interactions may influence biological activity, represents a central challenge. For example, studies on plant extracts, flavonoid oxidation products, and protein–antioxidant interactions suggest that antioxidant effects cannot be attributed to single compounds alone but rather emerge from dynamic and context-dependent systems (contributions 1, 17, and 23). This complexity necessitates more integrative, system-level approaches combining analytical chemistry with mechanistic biological models to better define structure–activity relationships and functional outcomes. In addition, critical aspects such as bioavailability, metabolism, and safety also remain insufficiently addressed. Comprehensive toxicological and pharmacokinetic evaluations are therefore recommended to establish safe and effective therapeutic windows, which are essential for the successful translation of antioxidant research into clinical and industrial applications.

Looking ahead, the field is on the right track to benefit from the continued integration of multidisciplinary approaches. Advances in high-resolution analytical techniques, increasingly sophisticated in vitro and in vivo models, and in silico predictive tools are expected to accelerate discovery and enable more precise characterisation of antioxidant mechanisms. In particular, the convergence of computational modelling with experimental validation, as demonstrated across several contributions, represents a promising pathway toward more rational, targeted, and potentially personalised antioxidant strategies.

## 4. Conclusions

This Topic highlights a field that has reached a high level of conceptual, methodological, and technological maturity. Research on natural antioxidants has evolved from descriptive assessments of chemical reactivity into an interdisciplinary science grounded in mechanistic understanding, biological relevance, translational intent, and sustainability. This progress provides a strong foundation for the next phase of development. Future advances will likely involve more predictive discovery strategies, improved formulation and delivery systems, and closer alignment between experimental findings and clinically relevant outcomes. In this context, the continued integration of advanced analytical chemistry, biologically meaningful models, and in silico tools will be important to fully harness the potential of natural antioxidants across diverse industrial and biomedical applications.

## Data Availability

No new data were created or analysed in this study. Data sharing is not applicable to this article.
